# Sixteen Polymorphic Simple Sequence Repeat Markers from Expressed Sequence Tags of the Chinese Mitten Crab *Eriocheir sinensis*

**DOI:** 10.3390/ijms11083035

**Published:** 2010-08-24

**Authors:** Xiang-Gang Gao, Hong-Jun Li, Yun-Feng Li, Li-Jun Sui, Bao Zhu, Yu Liang, Wei-Dong Liu, Chong-Bo He

**Affiliations:** 1 Liaoning Key Laboratory of Marine Fishery Molecular Biology, Liaoning Ocean and Fishery Science Institute, 50 Heishijiao St, Dalian, Liaoning 116023, China; E-Mail: xiangganggao@hotmail.com (X.-G.G.); oceanlhj@163.com (H.-J.L.); yfli2008@yahoo.cn (Y.-F.L.); cnliu51@tom.com (W.-D.L.); 2 College of Life Science, Liaoning Normal University, 100 Huanghe Rd. Dalian, Liaoning 116029, China; E-Mail: suilijun2006@126.com (L.-J.S.); swx04250zb@126.com (B.Z.); 3 College of Life Sciences and Biotechnology, Dalian Ocean University, 52 Heishijiao St, Dalian, Liaoning 116023, China; E-Mail: xiaoyu325000@hotmail.com

**Keywords:** Chinese mitten crab, Eriocheir sinensis, expressed sequence tags, simple sequence repeats

## Abstract

The Chinese mitten crab (*Eriocheir sinensis*) is an economically important aquaculture species in China. In this study, we developed and evaluated simple sequence repeat markers from expressed sequence tags of *E. sinensis*. Among the 40 wild *E. sinensis* individuals tested, 16 loci were polymorphic. The number of alleles per locus ranged from two to ten. The observed heterozygosity ranged from 0.0667 to 0.9667, whereas the expected heterozygosity ranged from 0.0661 to 0.9051. These markers have the potential for use in genetic studies of population structure and intraspecific variation in *E. sinensis*.

## 1. Introduction

The Chinese mitten crab (*Eriocheir sinensis*) is a native of East Asia that lives predominantly in freshwater but migrates seawards to breed. Economically, *E. sinensis* is an important cultured decapod crustacean in China because of its good taste. In order to protect genetic diversity and prevent population degradation, understanding the genetic diversity of *E. sinensis* is important. However, few studies have focused on the population structure of *E. sinensis* at the DNA level [1–3].

Microsatellites, also called simple sequence repeats (SSRs), are short tandem repeated sequences (1–6 bp) that are widely dispersed in eukaryotic genomes [4]. Because of high polymorphism, codominant inheritance, and even distribution throughout the genome, SSRs have been used extensively for studying genetic diversity and population structure in many species [5–7]. The expressed sequence tag (EST) represents part of the transcribed sequence and is an important resource for microsatellite and SNP screening [8,9]. In this study, we identified microsatellites from Chinese mitten crab ESTs and analyzed the polymorphisms present in the EST-SSRs.

## 2. Results and Discussion

A total of 16,961 Chinese mitten crab ESTs from the GenBank database (http://www.ncbi.nlm.nih.gov/Genbank/) were screened for microsatellites using the software MISA (http://pgrc.ipk-gatersleben.de/misa/) with the following parameters: at least eight repeats for di-, six repeats for tri-, five repeats for tetra-nucleotides, four repeats for penta-nucleotides, and three repeats for hexa-nucleotides. A total of 1,768 (10%) SSRs were derived from the 16,961 EST sequences. Analysis of these SSRs revealed that the dinucleotides (1189), hexanucleotides (616), and trinucleotides (615) were major motifs that accounted for 47%, 24%, and 24% of the total, respectively. CA/TG was the most frequent motif and accounted for 32%, followed by GA/TC (268, 11%). Forty SSR-containing ESTs that contained sufficient flanking sequences of good quality were chosen for polymerase chain reaction (PCR) analysis in *E. sinensis*. Primers were designed using the software Primer3 (http://biotools.umassmed.edu/bioapps/primer3_www.cgi). The results indicated that 20 (50%) of primer pairs could successfully amplify scorable products. The remaining primer pairs failed to amplify any PCR product, perhaps because the primer sequences spanned introns and/or contained mutations and/or indels (insertion or deletion). Among the 20 functional primer pairs, 16 loci showed polymorphism in the 40 *E. sinensis* individuals (Table 1), with the allele number ranging from 2 to 10. The observed heterozygosity ranged from 0.0667 to 0.9667, whereas the expected heterozygosity ranged from 0.0661 to 0.9051. Eight of the 16 loci (ESMS03, ESMS13, ESMS15, ESMS19, ESMS20, ESMS21, ESMS25 and ESMS35) departed from HWE (*P* < 0.05). This might be due to the limited sample size, and/or presence of overdominant selection, and/or a high degree of outbreeding. Further studies would be necessary for clarification. These polymorphic microsatellites derived from *E. sinensis* would be useful for population genetic structure analysis and genetic diversity assessment in crab populations and will facilitate breeding programs.

## 3. Experimental Section

Forty *E. sinensis* individuals were randomly collected from the Liaohe River in northeastern China. The leg muscles were removed from live individuals and stored in 80% ethanol until use. DNA was extracted following the traditional phenol/chloroform extraction method [9]. Each reaction was conducted in a 25 μL volume containing 50 ng of genomic DNA, 1 × PCR buffer, 1.5 mM MgCl_2_, 0.2 mM dNTPs, 200 nM of each primer, and 1U of Taq polymerase (Takara). The PCR program was as follows: initial denaturation for 5 min at 94 °C followed by 35 cycles of 30 s at 94 °C, 30 s at annealing temperature (see Table 1), and 30 s at 72 °C, with a final extension at 72 °C for 10 min. The amplified PCR products were separated on a 10% non-denaturing polyacrylamide gel at 280 V for 1–2 h, stained with ethidium bromide, and visualized under ultraviolet light. The genetic diversity indices, including observed and expected heterozygosities and tests for departures from Hardy- Weinberg equilibrium (HWE), were performed using POPGENE32 version 1.32 [10].

## Figures and Tables

**Table 1:** Characterization of EST-SSRs from the Chinese mitten crab (*Eriocheir sinensis*). *T*, annealing temperature; *N_A_*, number of alleles detected; *H_O_*, observed heterozygosity; *H_E_*, expected heterozygosity; *P*_HW_ < 0.05 indicates significant departure from Hardy-Weinberg equilibrium calculated for data from 40 *E. sinensis* individuals.

Locus (Acc. No.)	Repeat motif	Primer pair sequence (5′-3′)	Expected Size (bp)	*T* (°C)	*N_A_*	*H_O_*	*H_E_*	*P*_HW_
ESMS03 (FG359457)	(AC)_18_	F:CTGACGGCTACCTCCACTTC	223	53	7	0.966	0.848	0.000
		R:TTTCCTTCCATCCTGAGTCC				7	6	0
ESMS04 (FG359097)	(AGG)_6_	F:GCCTGCCTCAAGAATGGGTT	133	62	3	0.066	0.066	0.998
		R:GGTTGGTCTCCAGGAAGTGAAT				7	1	3
ESMS05 (FG359055)	(TCA)_7_...	F:ACGATACCCAAAGCAGAGGAC	211	62	3	0.333	0.287	0.612
	(CCT)_6_	R:ATGATGACGGAGACGACGAA				3	6	1
ESMS07 (FL574574)	(ACT)_12_	F:GTCACCACTGCTGCTTCTGC	168	60	3	0.333	0.552	0.065
		R:ACATTTGACGGTGGGACTGC				3	0	7
ESMS11 (FG983239)	(AC)_10_	F:TAGAGGTGGAAGATACTAGATGG	246	57	3	0.466	0.424	0.504
		R:TTGGAGGGTGGTAGGTTGAT				7	9	3
ESMS13 (FG981455)	(AC)_15_	F:CGCACGGGAAATGGAACAGA	249	53	6	0.966	0.814	0.000
		R:GAGGCATTTGAAAAGATGAAGCAC				7	7	5
ESMS15 (FG982058)	(CCA)_6_	F:GTGAAAGGACGGACGTATTGA	217	62	3	0.133	0.243	0.012
		R:GGAGGAAGAGGAGTGCGAGT				3	5	8
ESMS16 (FG982584)	(CCA)_9_	F:ACTGATGCCTGACGAAGACTACCA	184	62	6	0.866	0.774	0.486
	(ACA)_8_	R:CCTTTATGCCTTTATTGACCGAGAC				7	0	7
ESMS17 (FG983201)	(CA)_13_	F:GTATCCACAAGAGCATAAAGCAA	183	57	3	0.200	0.187	0.906
		R:AGCCAAACCTGAGAACCACT				0	6	2
ESMS19 (FG360290)	(TG)_13_	F:CTGAAGGTTTGCCTCGTGTT	205	60	7	0.900	0.842	0.005
		R:GGTGAAATGGACCAAATGAC				0	9	0
ESMS20 (FG359986)	(TC)_35_	F:TTGCGGTATCTTGCGTCTCG	220	62	10	0.966	0.905	0.000
		R:ATGTACCACAGCAACGCCTC				7	1	0
ESMS21 (FG359967)	(AC)_37_	F:GCAAACGAACTGATAAGCAC	192	56	9	0.933	0.852	0.039
		R:CTTTATGTTCCCAGGTGATG				3	0	6
ESMS24 (FG358074)	(CAC)_6_	F:CTTATCTCAGCGATGATTTGC	239	62	2	0.100	0.096	0.745
		R:AGCAGTGCCTGGTTTGTATT				0	6	5
ESMS25 (FG360197)	(TG)_17_	F:AACAGTTTGTAAGGTTCAGCAC	203	56	7	0.966	0.833	0.020
		R:TAGGGTGTAAATCCTCTGGC				7	9	5
ESMS26 (GE339913)	(AC)_17_...	F:ACGCACAAAGGCAACAAACTG	153	62	2	0.333	0.333	0.999
	(CGCA)_5_	R:AGGAAACGGCTGGCGAGACAA				3	3	7
ESMS35 (GE340314)	(GAG)_8_	F:TTGCCGAGAAGATCGCTTTGG	184	62	5	0.266	0.587	0.005
		R:GCCCGTCGCAGATACTGGTTT				7	0	8
